# A Universal System of CRISPR/Cas9-Mediated Gene Targeting Using All-in-One Vector in Plants

**DOI:** 10.3389/fgeed.2020.604289

**Published:** 2020-11-25

**Authors:** Ayako Nishizawa-Yokoi, Masafumi Mikami, Seiichi Toki

**Affiliations:** ^1^Plant Genome Engineering Research Unit, Institute of Agrobiological Sciences, National Agriculture and Food Research Organization (NARO), Tsukuba, Japan; ^2^Precursory Research for Embryonic Science and Technology (PRESTO), Japan Science and Technology Agency (JST), Kawaguchi, Japan; ^3^Graduate School of Nanobioscience, Yokohama City University, Yokohama, Japan; ^4^Kihara Institute for Biological Research, Yokohama City University, Yokohama, Japan

**Keywords:** gene targeting, homologous recombination, CRISPR/Cas9, genome editing, rice

## Abstract

Homologous recombination-mediated genome editing, also called gene targeting (GT), is an essential technique that allows precise modification of a target sequence, including introduction of point mutations, knock-in of a reporter gene, and/or swapping of a functional domain. However, due to its low frequency, it has been difficult to establish GT approaches that can be applied widely to a large number of plant species. We have developed a simple and universal clustered regularly interspaced short palindromic repeats (CRISPR)/CRISPR-associated protein 9 (Cas9)-mediated DNA double-strand break (DSB)-induced GT system using an all-in-one vector comprising a CRISPR/Cas9 expression construct, selectable marker, and GT donor template. This system enabled introduction of targeted point mutations with non-selectable traits into several target genes in both rice and tobacco. Since it was possible to evaluate the GT frequency on endogenous target genes precisely using this system, we investigated the effect of treatment with Rad51-stimulatory compound 1 (RS-1) on the frequency of DSB-induced GT. GT frequency was slightly, but consistently, improved by RS-1 treatment in both target plants.

## Introduction

Genome editing techniques have come to be required for both the development of basic research and plant molecular breeding in recent years. Targeted mutagenesis using sequence-specific nucleases (SSNs) such as transcription activator-like effector nucleases (TALENs) and the clustered regularly interspaced short palindromic repeats (CRISPR)/CRISPR-associated protein 9 (Cas9) is one of the genome editing techniques that has become available for several plant species and crops (Voytas, 2013; Puchta, 2017; Vats et al., 2019). However, it is difficult to deliberately introduce a desired mutation into target locus using this method because the mutations occur randomly in the process of repair *via* the non-homologous end joining (NHEJ) pathway of DNA double-strand breaks (DSBs) induced by SSNs. On the other hand, homologous recombination (HR)-mediated gene targeting (GT) allows precise genome engineering (the introduction of nucleotide substitutions, swapping of functional domains, and knock-in of reporter genes, etc.) of endogenous target genes *via* “copy and paste” of sequences from a repair template. This approach is essential to create gain-of-function-type mutants. However, establishing a universal GT method that can be applied to a large number of plant species remains a challenge.

It is well-known that the frequency of GT in higher plants is very low (Hrouda and Paszkowski, 1994; Gallego et al., 1999; Puchta, 2002). The positive–negative selection method has been used widely to enrich rare GT cells at least in rice (Terada et al., 2002; Yamauchi et al., 2009; Nishizawa-Yokoi et al., 2015). Using this method, a positive selection marker expression cassette is introduced into the target locus together with the desired mutations. Accordingly, we have used the *piggyBac* transposon, which transposes without leaving a footprint at the excised site, to allow precise marker elimination from a GT locus in rice (Nishizawa-Yokoi et al., 2015). We succeeded in introducing targeted point mutations or reporter genes into multiple target genes *via* GT with positive–negative selection and subsequent excision of the positive selection marker from the target locus using *piggyBac* transposition. However, such positive–negative selection has not been applied successfully to the selection of GT cells in higher plants other than rice.

The induction of SSN-mediated DSBs at specific target loci has been explored as a major strategy for the improvement of GT frequency in several plant species (Fauser et al., 2012, 2014; Cermák et al., 2015; Gil-Humanes et al., 2017; Miki et al., 2018). In almost all of these studies, GT cells have been enriched from a large number of non-GT cells using antibiotic, herbicide, and/or visible selection, resulting from the introduction of targeted point mutations conferring resistance to herbicide, knock-in of antibiotic resistance, or the introduction of a reporter gene into a target gene *via* GT. Thus, GT frequency is difficult to evaluate accurately using this system because the total number of cells transfected with the GT template is unclear.

On the other hand, various kinds of small-molecule compounds that have been developed to modulate DSB repair pathways (inhibit NHEJ or activate HR) have been utilized to improve the frequency of CRISPR/Cas9-mediated GT in mammalian cells (Danner et al., 2017; Hengel et al., 2017). It has been reported that Rad51-stimulatory compound 1 (RS-1) led to an increase in the CRISPR/Cas9- and TALEN-mediated knock-in frequency in rabbit, whereas SCR7 (a DNA ligase 4 inhibitor) had minimal effect (Jayathilaka et al., 2008; Pinder et al., 2015; Song et al., 2016).

We reveal here that a simple and easy-to-use CRISPR/Cas9-mediated GT method can introduce point mutations with a non-selectable trait into several target genes in rice and tobacco using an all-in-one GT vector carrying CRISPR/Cas9, a selectable marker gene, and a GT template. Furthermore, we evaluated the GT frequency on several endogenous target genes accurately using this method and investigated the effect of RS-1 on the frequency of GT with an all-in-one vector in rice and tobacco.

## Materials and Methods

### Plant Materials

Rice (*Oryza sativa* L. cv. Nipponbare) calli were induced from mature seeds as described previously (Saika and Toki, 2010) and were used for *Agrobacterium*-mediated transformation. Tobacco (*Nicotiana tabacum* L. cv. Petit Havana, SR-1) plants were grown in soil in a greenhouse (16 h light/8 h dark) at 21°C, and mature leaves of 3- or 4-week-old plants were used for *Agrobacterium*-mediated transformation.

### Vector Construction

All-in-one GT vectors are based on the CRISPR/Cas9 expression vectors described in a previous study as pZH_OsU6sgRNA_SpCas9-wt (Endo et al., 2019) or pZH_OsU6gRNA_MMCas9 (Mikami et al., 2015) for rice and pDe_Cas9_KAN (Fauser et al., 2014) for tobacco. The 20-nt annealed oligonucleotide pairs for the target sequences shown in Supplementary Table 1 were cloned into the *Bbs*I site of the single guide RNA (sgRNA) expression vector described as pU6gRNA-oligo (Mikami et al., 2015) for rice and pEn-Chimera (Fauser et al., 2014), pMR217, and pMR218 (Ritter et al., 2017) for tobacco, respectively.

An all-in-one GT vector for the introduction of point mutations into the *OsALS* gene was constructed as follows. The GT donor template (683 bp) containing a partial *OsALS* coding sequence with W548L/S627I mutations was amplified by PCR with the vector used in our previous study (Endo et al., 2007) using the primers shown in Supplementary Table 1. Two sgRNA expression cassettes (OsU6::sgOsALS_W548 and OsU6::sgOsALS_S627) were amplified by PCR using the primers shown in Supplementary Table 1. Three DNA fragments of the GT donor, OsU6::sgOsALS_W548, and OsU6::sgOsALS_S627 were introduced simultaneously into the *Asc*I site of pZH_OsU6sgRNA_SpCas9-wt by an in-fusion reaction (Takara). An all-in-one GT vector for the modification of *OsCly1* gene was constructed as follows. The sgRNA expression cassette (OsU6::sgCly1) was digested and ligated into the *Asc*I/*Pac*I site in pZH_OsU6gRNA_MMCas9. To insert the GT donor template into the vector, an artificially synthesized DNA fragment (1,001 bp) containing a partial *OsCly1* coding sequence with mutations in the miR172 targeting site was cloned into the *Asc*I site in pZH_OsU6sgOsCly1_MMCas9. For construction of an all-in-one GT vector in tobacco, the sgRNA expression cassettes (AtU6::sgNtALS in pEn-Chimera, AtU6::sgNtEPSPS_T176 in pMR217, and AtU6::sgNtEPSPS_P180 in pMR218) were generated *via* a cut-ligation reaction with *Bbs*I as described above. Single (for *NtALS*) or double sgRNA modules (for *NtEPSPS*) were combined with pDe_Cas9_KAN using a Gateway LR reaction (Thermo Fisher Scientific). GT donor templates for the modification of *NtALS* (1,040 bp DNA fragments containing a partial *NtALS-B* coding sequence with W568L/S647I mutations) or *NtEPSPS* (1,018 bp DNA fragments carrying a partial *NtEPSPS-B* with T176I/P180S mutations) were synthesized artificially and introduced into the *Pac*I site of pDe_Cas9_AtU6sgNtALS or pDe_Cas9_AtU6sgNtEPSPSx2, respectively.

### *Agrobacterium*-Mediated Transformation

All-in-one GT vectors were transformed into rice and tobacco by *Agrobacterium*-mediated transformation as described previously (Horsch et al., 1985; Toki et al., 2006). Rice calli were immersed and shaken gently in AAM medium with 25 μM RS-1 or 0.05% dimethyl sulfoxide (DMSO) as a control for 30 min before *Agrobacterium* infection. Transgenic rice calli were selected on medium N6D containing 50 mg/L hygromycin, 25 mg/L meropenem (Wako Pure Chemical Industries), and 25 μM RS-1 or 0.05% DMSO for 2 weeks at 33°C. Hygromycin-resistant calli were transferred to N6D containing 50 mg/L hygromycin and 25 mg/L meropenem without RS-1 or DMSO and cultured for 2 more weeks. To evaluate GT frequency targeting the *OsALS* locus without CRISPR/Cas9-mediated DSB induction, a vector harboring an 8-kb fragment of genomic DNA encoding the 5′ truncated *OsALS* gene carrying W548L and S627I mutations was transformed into rice calli by *Agrobacterium*-mediated transformation (Horsch et al., 1985; Toki et al., 2006). Transgenic rice calli were cultured on N6D medium containing 25 mg/L meropenem, 0.75 μM herbicide bispyribac (BS), and 25 μM RS-1 or 0.05% DMSO for 3 weeks. BS-tolerant calli were transferred to N6D medium containing 25 mg/L meropenem and 0.75 μM BS without RS-1 or DMSO and cultured for a further 2 weeks.

Tobacco leaf discs were punched from surface-sterilized leaves with a cork borer (6 mm in diameter) and treated with 25 μM RS-1 or 0.05% DMSO as a control for 30 min in Murashige and Skoog (MS) medium (Murashige and Skoog, 1962). Leaf discs were then inoculated with *Agrobacterium* strain EHA105 (Hood et al., 1993) harboring an all-in-one GT vector. After 3 days of co-cultivation with *Agrobacterium*, leaf discs were transferred to regeneration medium with 100 mg/L kanamycin, 25 mg/L meropenem, and 25 μM RS-1 or 0.05% DMSO for 2 weeks and cultured under a 16-h light/8-h dark cycle at 27°C. Kanamycin-resistant calli were transferred to fresh medium with 100 mg/L kanamycin and 25 mg/L meropenem without RS-1 or 0.05% DMSO and cultured for 2 more weeks.

### Chemicals

RS-1 and the Lig4 inhibitor, SCR7, were purchased commercially (Cosmo Bio). A portion of these compounds as used in this study was synthesized as described previously (Jayathilaka et al., 2008; Greco et al., 2016).

### Screening of Gene Targeting Candidate Calli by Cleaved Amplified Polymorphic Sequence Analysis

Genomic DNA extracted from small pieces of clonally propagated hygromycin-resistant rice calli or kanamycin-resistant tobacco calli using Agencourt chloropure (Beckman Coulter) according to the manufacturer's protocol was subjected to cleaved amplified polymorphic sequence (CAPS) analysis. PCR amplifications were performed with KOD FX neo or KOD ONE (TOYOBO) using the primer sets shown in Supplementary Table 1. PCR products were digested with restriction enzyme *Mfe*I for *OsALS* and *NtALS-B, Xba*I for *OsCly1*, and *Hin*dIII for *NtEPSPS-B* and analyzed with MultiNA microchip electrophoresis system (Shimadzu).

PCR fragments derived from CAPS-positive calli or plants were cloned into pCR-BluntII-TOPO (Invitrogen) and subjected to sequence analysis using an ABI3130 sequencer (Applied Biosystems).

### Southern Blot Analysis

Genomic DNA was extracted from leaves of seedlings using the Nucleon Phytopure extraction kit (Cytiva) according to the manufacturer's protocol. Two micrograms of rice genomic DNA or 15 μg of tobacco genomic DNA was digested with the same restriction enzyme as that used in CAPS analysis and fractionated in a 0.8% agarose gel. Southern blot analysis was performed according to the digoxigenin (DIG) Application Manual (Sigma-Aldrich). Specific DNA probes for the *hpt, nptII*, and *OsCly1* locus were synthesized with a PCR DIG probe synthesis kit (Sigma-Aldrich) according to the manufacturer's protocol using the primers shown in Supplementary Table 1.

### Herbicide-Susceptibility Testing in Rice and Tobacco

Seeds of wild-type rice (Nipponbare) and T_1_ progeny of OsALS-GT RS-1_B were sown on 1/2 MS medium with or without 1.5 μM BS and were grown in a growth chamber at 27°C under a 16 h photoperiod. Seeds of wild-type tobacco (SR-1) and T_1_ progeny of NtALS_GT RS-1_B#1 were sown on 1/2 MS medium with or without 500 nM chlorsulfuron (CS) and grown in a growth chamber (16 h light/8 h dark) at 27°C.

## Results

### Establishing a CRISPR/Cas9-Mediated Gene Targeting Method With an All-in-One Vector in Rice

**Figure 1:**
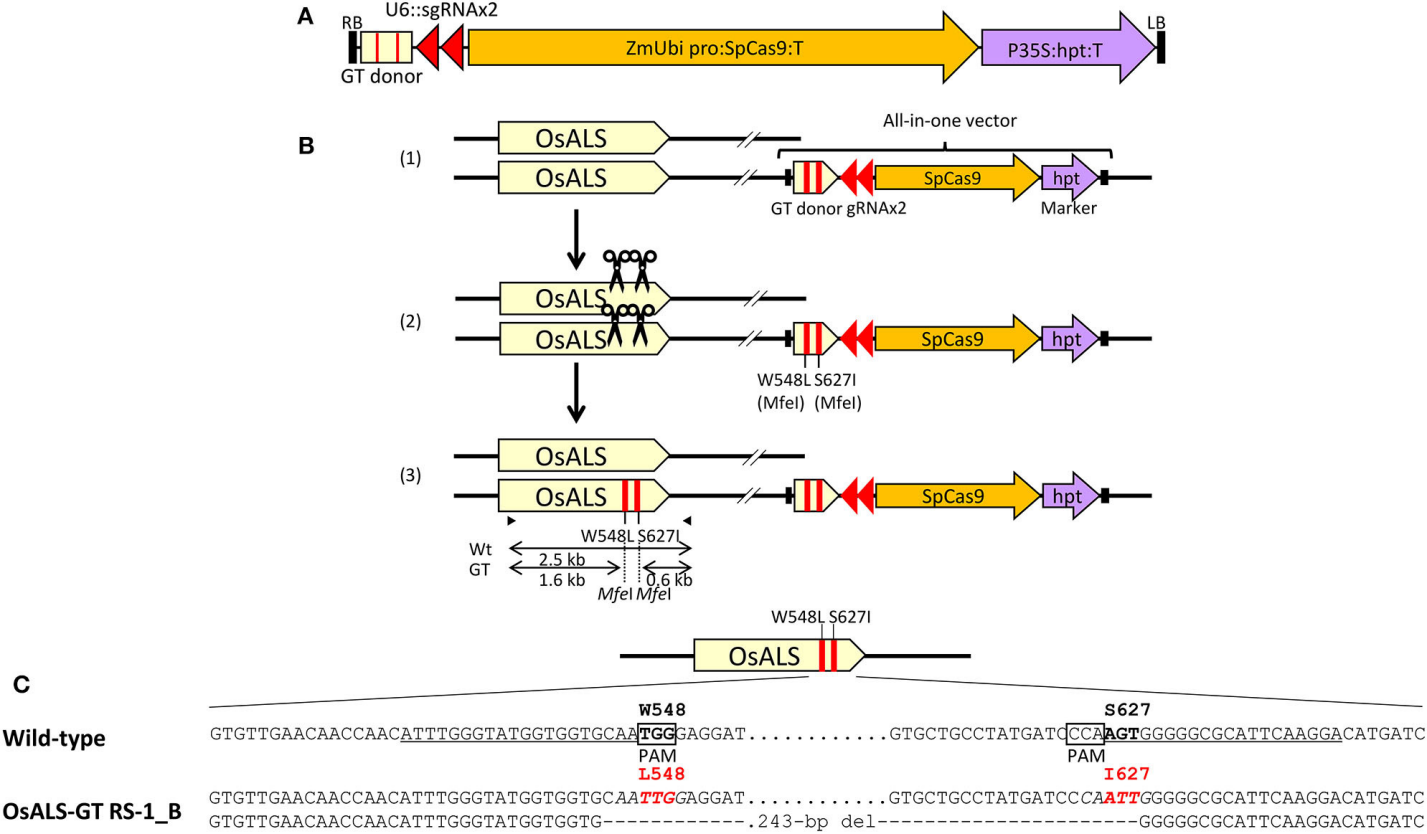
Introduction of point mutations into the rice *ALS* locus *via* clustered regularly interspaced short palindromic repeats (CRISPR)/CRISPR-associated protein 9 (Cas9)-mediated gene targeting (GT) with an all-in-one vector. **(A)** Schematic diagram of the all-in-one GT vector for modification of the rice acetolactate synthase (*OsALS*) locus. The vector carries two sgRNA expression units (OsU6::sgOsALS_W548 and OsU6::sgOsALS_S627), SpCas9 expression cassette (maize ubiquitin promoter::OsADH 5′UTR-SpCas9-SV40 NLS::terminator), selection marker (CaMV35S promoter::hpt::terminator), and GT donor template (ca. 700 bp) containing a partial *OsALS* coding sequence with W548L and S627I mutations. **(B)** CRISPR/Cas9-mediated GT strategy in rice. (1) The all-in-one GT vector was integrated into the host genome *via Agrobacterium*-mediated transformation. (2) Following the expression of CRISPR/Cas9 from the GT vector on the genome, a double-strand break (DSB) was induced at the target site during the selection of transgenic cells. (3) Repair of DSB *via* homologous recombination (HR) between the target gene and GT donor then introduces the desired mutations into the target gene. The primer sets used for cleaved amplified polymorphic sequence (CAPS) analysis that identify transgenic calli in which a GT event occurred at the *OsALS* locus are shown as black arrows. **(C)** Nucleotide sequences of the *OsALS* gene in wild-type (top) and regenerated plants from OsALS-GT RS-1_B calli (bottom). Target sites of sgRNA are underlined, W548L and S627I mutations *via* GT are in red, and the recognition sites of *Mfe*I (CAATTG) used for CAPS analysis are in italics.

**Figure 2:**
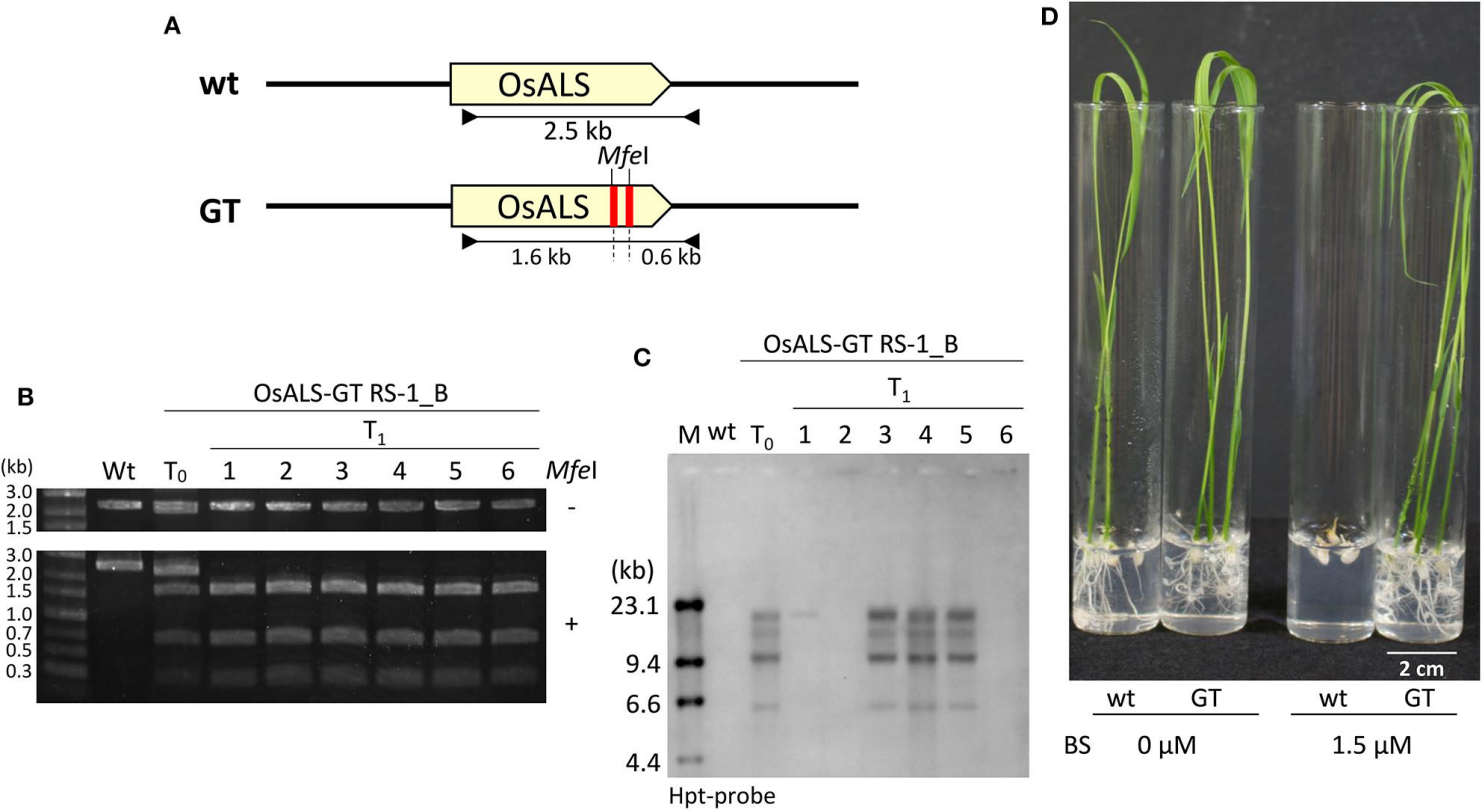
Functional analysis of modified *OsALS* gene in T_1_ progeny. **(A)** Genomic structure of wild-type *OsALS* locus (top) and the modified *OsALS* locus in T_1_ plants from OsALS-GT RS-1_B calli (bottom). The primer sets used for cleaved amplified polymorphic sequence (CAPS) analysis are shown as black arrows. **(B)** CAPS analysis with genomic DNA of wild type, T_0_ regenerated plants, and T_1_ progeny of OsALS-GT RS-1_B using *OsALS*-specific primers shown in **(A)**. PCR fragments were digested with (lower) or without (upper) *Mfe*I (*Mfe*I + and –, respectively). **(C)** Southern blot analysis with the hpt-specific probe using *Mfe*I-digested genomic DNA of wild type, T_0_ regenerated plants, and T_1_ progeny of OsALS-GT RS-1_B. **(D)** Herbicide bispyribac (BS)-tolerant phenotype of T_1_ plants of OsALS-GT RS-1_B.

Previously, we established a system to select infrequent GT cells by introducing two amino acid substitutions (W548L and S627I) conferring tolerance to the herbicide bispyribac sodium (BS) into the rice *acetolactate synthase* (*OsALS*) gene (Endo et al., 2007). The *ALS* gene has been used as a model target for development of a GT system in various plant species (Zhang et al., 2013; Nishizawa-Yokoi et al., 2015; Wolter et al., 2018). We also succeeded in introducing two amino acid substitutions (W548L/S627I) into the *OsALS* gene by GT with positive–negative selection (Nishizawa-Yokoi et al., 2015) rather than using herbicide selection. Thus, an all-in-one GT vector harboring two sgRNAs targeting the *OsALS* gene, Cas9, *hygromycin phosphotransferase* (*hpt*) gene expression cassettes, and a GT donor template (ca. 700 bp) carrying W548L and S627I, in which these point mutations generate recognition sites for the restriction enzyme *Mfe*I, CAATTG, was constructed and transformed into rice calli (Figures 1A,B). The desired point mutations were located on the protospacer adjacent motif (PAM) sequences following the DNA region targeted by SpCas9, indicating that the GT donor template on the vectors was not cleaved by the CRISPR/Cas9 system. To examine the cytotoxicity of RS-1 in rice calli, wild-type calli were cultured on medium containing 25 or 50 μM RS-1 for 2 weeks. No significant differences in the growth of calli were observed between the treatment groups with DMSO as a control, 25 or 50 μM RS-1 (Supplementary Figure 1). Therefore, 25 μM RS-1 was used for subsequent experiments. Rice calli transformed with an all-in-one GT vector were selected on medium containing hygromycin B, not herbicide BS, with or without RS-1, to investigate the ratio of GT cells to *Agrobacterium*-mediated transformed cells in rice. After a 2-week selection period, transgenic callus lines were cloned and further propagated for another 2 weeks. Genomic DNA was extracted from 960 callus lines each of the control (DMSO treatment) and 25 μM RS-1 treatment groups. CAPS analysis, i.e., PCR analysis coupled with *Mfe*I digestion, revealed that there were no GT positive callus lines from among 960 independent transgenic calli harboring all-in-one GT vector treated with DMSO (Table 1), while *Mfe*I-digested PCR fragments were detected in two independent callus lines, designated as OsALS-GT RS-1_A and B, among the 960 all-in-one GT vector transgenic calli treated with 25 μM RS-1, suggesting that, in these callus lines, W548L/S627I mutations were introduced into the *OsALS* locus by HR between the GT vector that could be integrated into the genome and the target locus. Thus, the frequency of GT calli to transgenic calli analyzed by CAPS was 0.21% (2/960) under treatment with RS-1 (Table 1). Sequencing analysis of PCR fragments derived from the *OsALS* locus in OsALS-GT RS-1_A and B revealed that the proportion of GT cells carrying a GT-modified *OsALS* locus within the transgenic calli analyzed was 8.3% (2/24) and 37.5% (9/24), respectively (Supplementary Table 2). These results suggest that transgenic cells with W548L/S627I mutations *via* GT and without these mutations at the *OsALS* locus existed in a chimeric state in a single callus clone. OsALS-GT RS-1_B calli were transferred to regeneration medium to obtain regenerated plants, and the *OsALS* locus of T_0_ regenerated plants was genotyped using CAPS and sequencing analysis. All regenerated plants (OsALS-GT RS-1_B#1-#24) from OsALS-GT RS-1_B analyzed (24/24, 100%) had an identical mutation pattern, which was the desired point mutation introduced *via* GT and the long deletion (243 bp) between the two target sequences of CRISPR/Cas9 in each *OsALS* allele, respectively (Figure 1C). Furthermore, we obtained T_1_ plants from self-pollinating regenerated plants (OsALS-GT RS-1_B#1 and #2) of the OsALS-GT RS-1_B line and conducted genotyping of the *OsALS* gene and the BS-sensitivity test. We found that all progeny plants from OsALS-GT RS-1_B#1 T_0_ plants carried homozygous point mutations *via* GT in the *OsALS* gene (Table 2; Figures 2A,B). In another line, OsALS-GT RS-1_B#2, 96.8% (30/31) of progeny plants were homozygous mutants harboring the desired point mutations *via* GT in the *OsALS* gene, while only one plant (3.2%) was a heterozygous mutant carrying GT-mediated point mutations and the CRISPR/Cas9-mediated deletion in each allele of *OsALS* (Table 2). These results indicate that defects in the *OsALS* gene might cause a sterility phenotype in rice even when its mutations are introduced into one allele; in other words, it was likely that the loss of the *OsALS* gene induced the defect of gametogenesis. What is more important is that progeny plants carrying W548L/S627I mutations in the *OsALS* gene and lacking all-in-one vectors could be obtained by genetic segregation *via* self-crossing of regenerated plants (Figures 2B,C). These T_1_ plants showed a herbicide BS-tolerant phenotype compared with wild-type plants (Figure 2D). To prove the effect of treatment with RS-1 on GT efficiency in rice, we evaluated GT frequency by introducing W548L/S627I substitutions into the *OsALS* gene without CRISPR/Cas9-mediated DSB induction. Rice calli were transformed with the vector harboring an 8-kb fragment of genomic DNA encoding the 5′ truncated *OsALS* gene carrying W548L/S627I mutations (Endo et al., 2007) and cultured on medium containing BS with or without RS-1 for 3 weeks. We obtained 38 and 71 callus lines of 6,561 and 6,289 transgenic calli treated with DMSO and RS-1, respectively (Table 3). CAPS analysis revealed that 35 and 64 callus lines treated with DMSO and RS-1, respectively, were true GT lines carrying two point mutations in the *OsALS* gene *via* GT. The proportion of true GT callus lines to transgenic calli was 0.5 and 1.0% under treatment with DMSO and RS-1, respectively (Table 3). These data provide support for the positive effects of RS-1 on GT.

**Table 1:** Summary of gene targeting (GT) experiments targeting OsALS locus using an all-in-one GT vector.

**Experiments**	**Treatment**	**No. of hygromycin-resistant calli analyzed**	**No. of calli with W548L/S627I mutations on OsALS**	**GT frequency (%)**
A	DMSO	576	0	0
	25 μM RS-1	576	1^*^	0.17
B	DMSO	384	0	0
	25 μM RS-1	384	1^**^	0.26
Total	DMSO	960	0	0
	RS-1	960	2	0.21

*GT positive callus lines were designated as follows*:

* *OsALS-GT RS-1_A*;

** *OsALS-GT RS-1_B*.

**Table 2:** Inheritance of target mutations and segregation of an all-in-one gene targeting (GT) vector in T_1_ plants.

**Line no. analyzed**	**No. of T_**1**_ plants**	**No. of progeny plants carrying W548L/S627I mutations via GT in** ***OsALS*** **locus**						**The existence of all-in-one vector**	
		**wild-type**	**%**	**Hetero**	**%**	**Homo**	**%**	**+**	**–**
OsALS-GT RS-1_B#1	36	0	0	0	0	36	100	34	2
OsALS-GT RS-1_B#2	31	0	0	1	3.2	30	96.8	30	1

**Table 3:** Summary of gene targeting (GT) experiments targeting the *OsALS* locus without clustered regularly interspaced short palindromic repeats (CRISPR)/CRISPR-associated protein 9 (Cas9)-mediated double-strand break (DSB) induction.

**Experiments**	**Treatment**	**No. of *Agrobacterium* infected calli**	**No. of BS- resistant calli**	**True GT calli**	
				**No. of calli**	**(%)**
A	DMSO	843	2	2	0.24
	25 μM RS-1	688	10	9	1.31
B	DMSO	1,209	2	2	0.17
	25 μM RS-1	1,352	11	9	0.67
C	DMSO	1,966	11	11	0.56
	25 μM RS-1	1,846	23	22	1.19
D	DMSO	2,543	23	20	0.79
	25 μM RS-1	2,403	27	24	1.00
Total	DMSO	6,561	38	35	0.53
	25 μM RS-1	6,289	71	64	1.02

**Figure 3:**
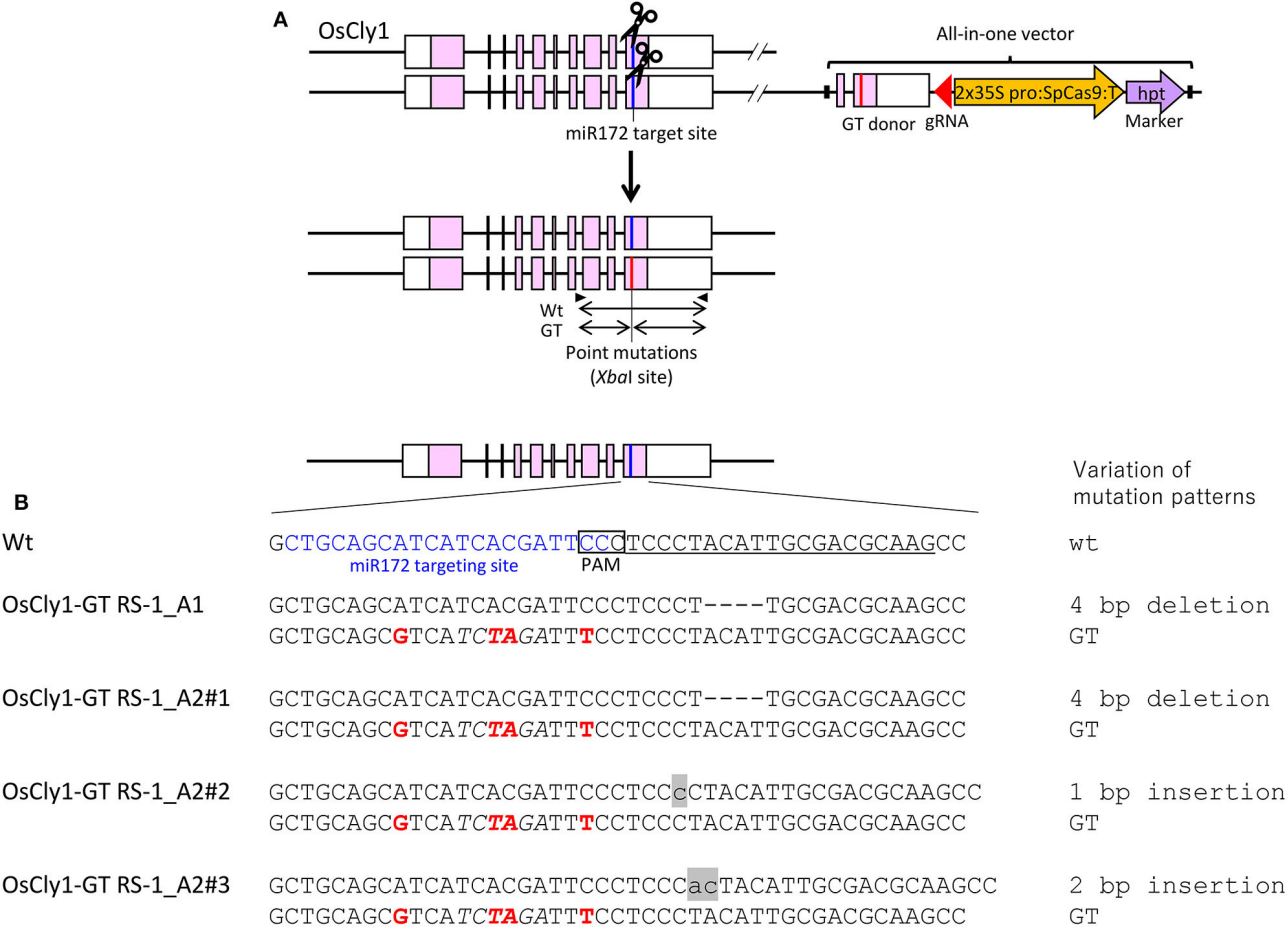
Modification of the miR172 targeting site of the rice *Cly1* gene *via* clustered regularly interspaced short palindromic repeats (CRISPR)/CRISPR-associated protein 9 (Cas9)-mediated gene targeting (GT) with an all-in-one vector. **(A)** Genomic structure of the *OsCly1* gene locus and schematic of the all-in-one GT vector. Black arrows indicate the primer set for cleaved amplified polymorphic sequence (CAPS) analysis to identify transgenic cells carrying a GT-modified *OsCly1* gene. **(B)** Nucleotide sequences of the *OsCly1* gene in wild-type (top) and regenerated plants from OsCly1-GT RS-1_A1, A2#1, A2#2, A2#3 calli. Target sites of sgRNA are underlined, point mutations introduced by GT are in red, recognition sites of *Xba*I (TCTAGA) used for CAPS analysis are in italics, miR172 targeting site are in blue, and insertional mutations are noted in lowercase letters and gray highlights.

To confirm that CRISPR/Cas9-mediated GT with an all-in-one vector is a universal approach in rice, we applied this approach to modify the microRNA miR172 targeting site of the rice *cleistogamy 1* (*OsCly1*) gene (Chen, 2004; Nair et al., 2010)—a model gene for GT experiments in rice in our hands (Nishizawa-Yokoi et al., 2015). The miR172 targeting site of the *OsCly1* gene was edited by GT, producing a recognition site for the restriction enzyme *Xba*I, allowing isolation of GT cells by CAPS analysis (wild-type: CTGCAGCATCATCACGATTCC, GT: CTGCAGCgTCA*TCtaGA*TTtC, *Xba*I site in italics), with no change to amino acid sequences (Figure 3A). CAPS analysis revealed that each of two independent lines of 768 hygromycin-resistant calli were GT lines, with treatment with DMSO (OsCly1-GT DSMO_B1 and B2) as a control or RS-1 (OsCly1-GT RS-1_A1 and A2), respectively, indicating that the GT frequency was 0.26% in each experimental group (Table 4). Regenerated plants were obtained from these GT lines, and the genotype of the *OsCly1* gene was analyzed by CAPS and sequencing analysis. Out of 24 regenerated plants from OsCly1-GT RS-1_A1 and A2 callus lines, nine (40%) and 12 (50%), respectively, were true GT plants containing the desired GT-mediated mutations and CRISPR/Cas9-mediated indels in each allele of the *OsCly1* gene, while all regenerated plants from OsCly1-GT DSMO_B1 and B2 callus lines lacked GT-mediated point mutations in the *OsCly1* locus (0/24) (Supplementary Table 3). All GT plants from the OsCly1-GT RS-1_A1 line (9/9) contained an identical mutation pattern (mutations *via* GT/4 bp deletion) in the *OsCly1* gene (Figure 3B), while different mutation patterns were observed between individual GT plants from the OsCly1-GT RS-1_A2 line. These different mutation patterns were classified into three groups (mutations *via* GT/4 bp deletion, mutations *via* GT/1 bp insertion, and mutations *via* GT/2 bp insertion) (Figure 3B). Furthermore, we verified that progeny plants carrying biallelic GT-mediated point mutations in the *OsCly1* gene and lacking the all-in-one vector could be obtained *via* genetic segregation (Supplementary Figure 2).

**Table 4:** Summary of gene targeting (GT) experiments targeting miR172 site of *OsCly1* gene using an all-in-one GT vector.

**Experiments**	**Treatment**	**No. of hygromycin-resistant calli analyzed**	**No. of calli with mutations on the microRNA targeting site of *OsCly1* gene**	**GT frequency (%)**
A	DMSO	384	0	0
	25 μM RS-1	384	2^*^	0.52
B	DMSO	384	2^**^	0.52
	25 μM RS-1	384	0	0
Total	DMSO	768	2	0.26
	25 μM RS-1	768	2	0.26

*GT positive callus lines were designated as follows*:

* *OsCly1-GT RS-1_A1 and A2*;

** *OsCly1-GT DMSO_B1 and B2*.

### Precise Gene Editing *via* Gene Targeting With All-in-One Vector in Tobacco

**Figure 4:**
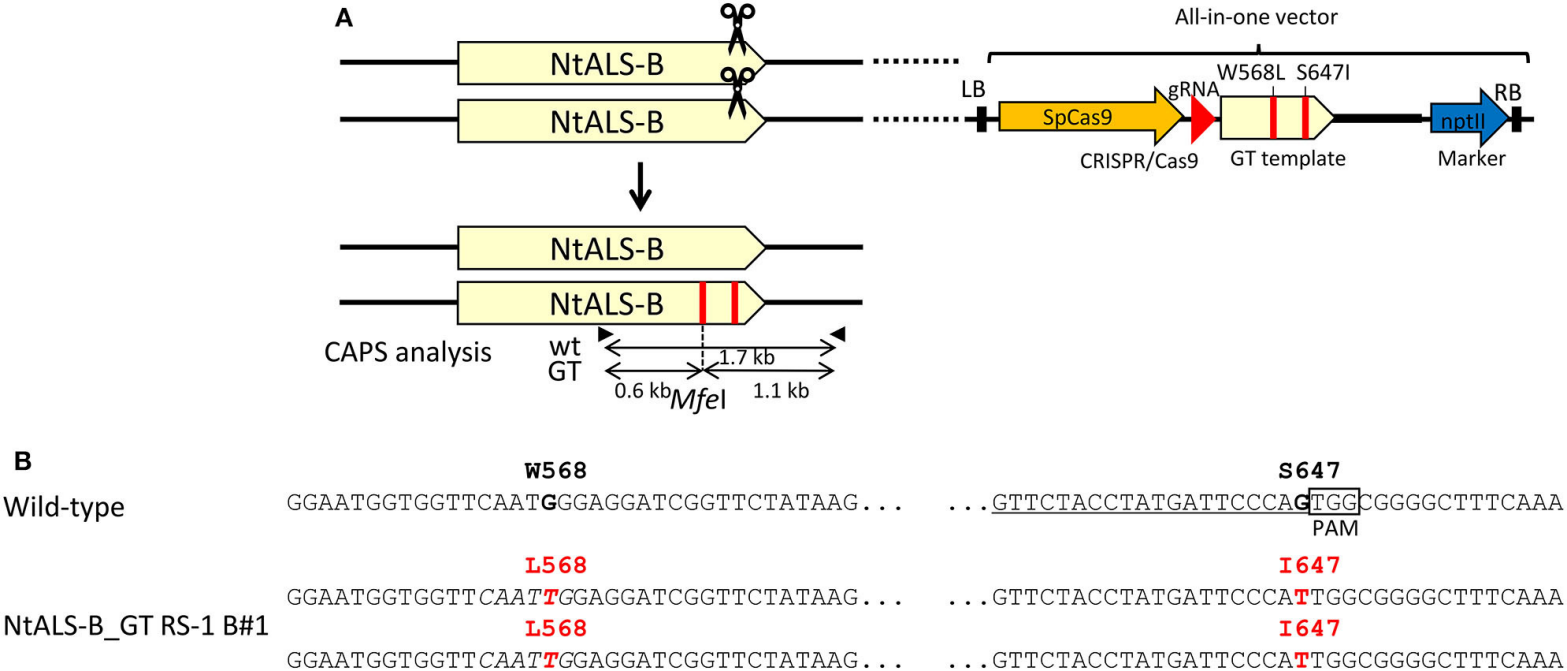
Biallelic gene targeting (GT) event found in the tobacco *ALS* gene (*NtALS-B*) locus in T_0_ plants *via* clustered regularly interspaced short palindromic repeats (CRISPR)/CRISPR-associated protein 9 (Cas9)-mediated GT with an all-in-one vector. **(A)** (Top) Genomic structure of the wild-type *NtALS* locus and schematic of the all-in-one GT vector targeting the *NtALS-B* locus. (Bottom) Genomic structure of the modified *NtALS* locus in GT plants. Black arrows indicate the primer set for cleaved amplified polymorphic sequence (CAPS) analysis to identify GT lines. **(B)** Nucleotide sequences of *NtALS-B* gene in wild-type (top) and regenerated plants from NtALS-B_GT RS-1 B#1. Target sites of sgRNA are underlined, point mutations introduced by GT are in red, and recognition sites of *MfeI*I (CAATTG) used for CAPS analysis are in italics.

To demonstrate that this CRISPR/Cas9-mediated GT approach with an all-in-one vector can be applied to other plant species, we designed an experiment aimed at substituting two amino acids (W568L/S647I) conferring tolerance to the herbicide CS in tobacco ALS-B (*NtALS-B*) using an all-in-one vector. A vector carrying an sgRNA targeting region proximal to S647 in the *NtALS-B* gene, Cas9, nptII expression cassettes, and GT donor template 1.0 kb *NtALS-B* coding region with mutations (W568L/*Mfe*I site and S647I) was introduced into tobacco leaf discs with or without RS-1 by *Agrobacterium*-mediated transformation (Figure 4A). After coculture with *Agrobacterium*, tobacco leaf discs were transferred to the selection medium containing kanamycin and DMSO or RS-1 and were cultured for 2 weeks. Kanamycin-resistant calli were propagated for another 2 weeks on selection medium without DMSO or RS-1. CAPS analysis revealed that 0.07% (1/1,357; Line no. NtALS_GT DMSO_C) and 0.22% (3/1,382; Line no. NtALS_GT RS-1_A1, A2, and B) of transgenic calli treated with DMSO and RS-1, respectively, harbored a GT-modified *NtALS-B* gene (Table 5). Regenerated plants obtained from CAPS-positive calli were subjected to CAPS and sequencing analysis to confirm the GT-mediated introduction of W568L and S647I mutations in the *NtALS-B* gene. In CAPS analysis, *Mfe*I-digested fragments were detected in 6.9% (4/58, NtALS_GT RS-1_B#1 to B#4) and 7.8% (4/51, NtALS_GT_DMSO_C#1 to C#4) of T0 plants regenerated from NtALS_GT RS-1_B and DMSO_C callus lines, respectively, but not from NtALS_GT RS-1_A1 and A2. Sequencing analysis of PCR fragments from CAPS-positive plants (NtALS_GT RS-1_B#1-B#4 and NtALS_GT_DMSO_C#1-C#4) showed that W568L (*Mfe*I recognition site) and S647I mutations had been introduced into the targeted *NtALS-B* gene in the biallelic state in NtALS_GT_RS-1_B#1-B#4 or in the chimeric state in DMSO_C#1–C#4, respectively. However, in plants NtALS_GT_RS-1_B#2–B#4 and DMSO_C#1–C#4, unexpected mutations (insertion or deletion) also occurred at the CRISPR/Cas9 target region, suggesting that CRISPR/Cas9 recognized and digested again at the target sequence even after the introduction of S647I mutations *via* GT (Supplementary Figure 3), whereas only one plant (NtALS_GT RS-1_B#1) carried biallelic W568L/S647I mutations in the *NtALS-B* gene without CRISPR/Cas9-mediated dispensable mutations (Figure 4B, Supplementary Figure 3).

**Table 5:** Summary of gene targeting (GT) experiments targeting *NtALS-B* gene using an all-in-one GT vector.

**Experiments**	**Treatment**	**No. of Kanamycin-resistant calli analyzed**	**No. of CAPS-positive callus lines**	**%**
A	DMSO	661	0	0
	25 μM RS-1	631	2^*^	0.32
B	DMSO	408	0	0
	25 μM RS-1	463	1^**^	0.22
C	DMSO	288	1^***^	0.35
	25 μM RS-1	288	0	0
Total	DMSO	1,357	1	0.07
	25 μM RS-1	1,382	3	0.22

*GT positive callus lines were designated as follows*:

* *NtALS_GT RS-1_A1 and A2*;

** *NtALS_GT RS-1_B*;

*** *NtALS_GT DMSO_C*.

**Figure 5:**
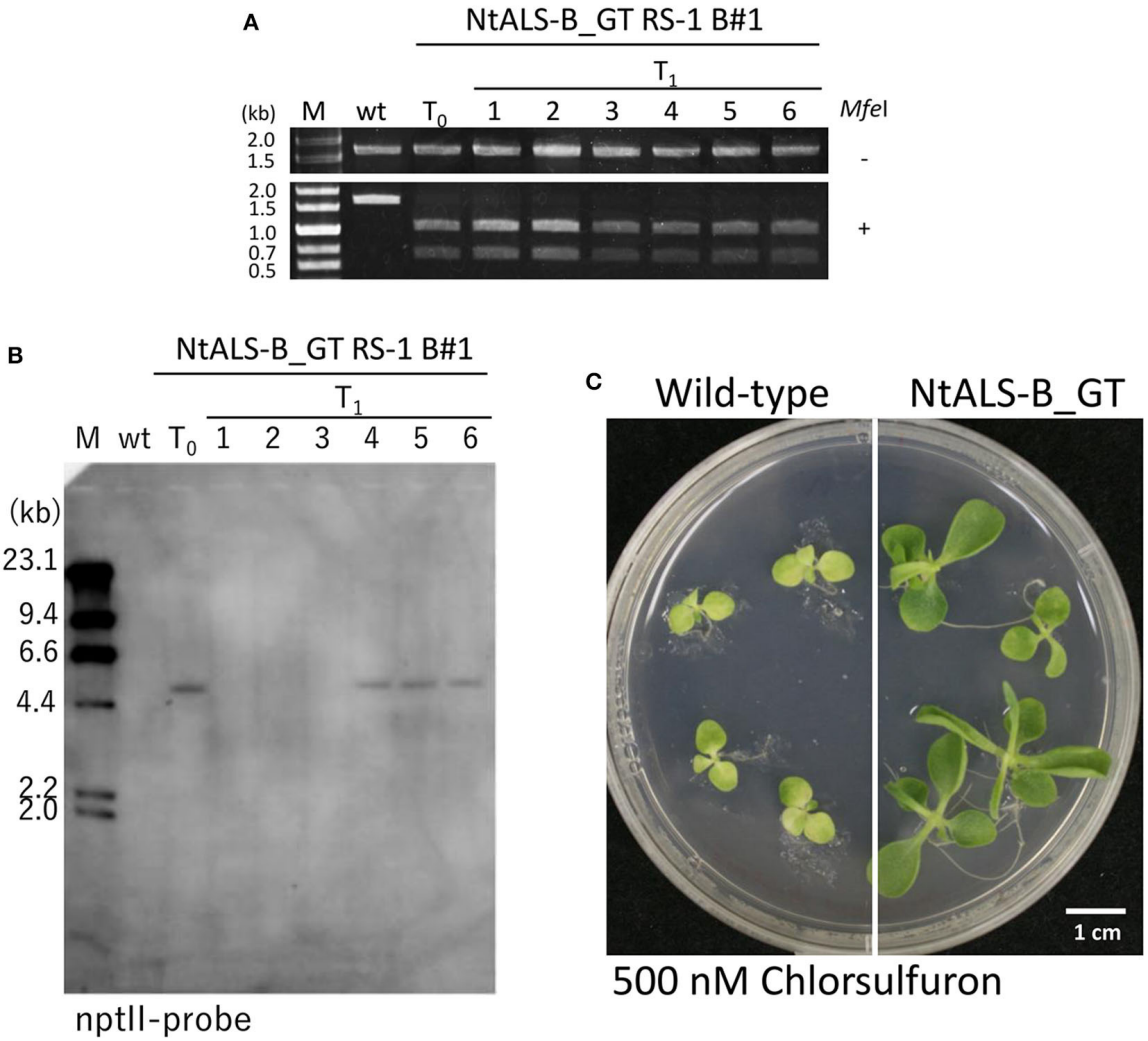
Modified *NtALS-B* gene with W568L/S647I mutations confers a chlorsulfuron (CS)-tolerant phenotype in T_1_ progeny of NtALS-B_GT RS-1 B#1. **(A)** Cleaved amplified polymorphic sequence (CAPS) analysis with genomic DNA of wild type, T_0_ regenerated plants, and T_1_ progeny of NtALS-B_GT RS-1_B#1 using *NtALS* specific primers shown in Figure 4A. PCR fragments were digested with (lower) or without (upper) *Mfe*I (*Mfe*I + and –, respectively). **(B)** Southern blot analysis with an nptII-specific probe using wild type, T_0_, and T_1_ plants of NtALS-B_GT RS-1 B#1. Line nos. 1–3 of T_1_ plants were found to be transgene-null segregants. **(C)** Herbicide CS-tolerant phenotype of T_1_ plants of NtALS-B_GT RS-1 B#1.

To confirm that W568L/S647I amino acid substitutions introduced *via* GT were stable and heritable, we obtained T_1_ progenies from NtALS_GT RS-1_B#1 regenerated plants with homozygous desired mutations in the *NtALS-B* gene. Sequencing analysis revealed that all progeny plants harbored a GT-modified *NtALS-B* gene with homozygous W568L/S647I mutations (34/34); however, additional mutations were also generated by CRISPR/Cas9 in 37.5% of T_1_ progenies (Table 6). We found diverse patterns (insertions or deletions) of additional mutation by CRISPR/Cas9 at the *NtALS-B* gene target site in siblings of NtALS_GT RS-1_B#1 T_1_ plants (Supplementary Figure 4). Furthermore, the all-in-one vector segregated out in 44.1% of progeny plants with homozygous W568L/S647I mutations and without additional CRISPR/Cas9-mediated mutations (Figures 5A,B). We also confirmed that the introduction of W568L/S647I mutations in *NtALS-B via* GT conferred a CS-tolerant phenotype on tobacco plants (Figure 5C).

**Table 6:** Inheritance of target mutations *via* gene targeting (GT) into NtALS_GT RS-1_B#1 progenies.

**No. of plants analyzed**	**No. of plants with W548L via GT in *NtALS-B***	**%**	**No. of plants with mutations via CRISPR/Cas9 at S647 in *NtALS-B***	**%**	**No. of null segregants of all-in-one vector**	**%**
34	34	100	9	37.5	15	44.1

The next target for precise modification *via* GT in tobacco was the gene encoding endogenous *3-phosphoshikimate 1-carboxyvinyltransferase* (*EPSPS*), a well-known target of the herbicide glyphosate that catalyzes an essential step in the shikimate pathway common to aromatic amino acid biosynthesis (Steinrücken and Amrhein, 1980). Yu et al. (2015) showed that two amino acid substitutions [T102I and P106S (TIPS)] in a conserved region of the *EPSPS* gene led to high-level glyphosate resistance in goose grass (*Eleusine indica*). Thus, we attempted to introduce TIPS (T176I and P180S) amino acid substitutions into tobacco *EPSPS-B* (*NtEPSPS-B*) using a CRISPR/Cas9-mediated GT approach (Supplementary Figures 5A,B). Tobacco leaf discs were infected with *Agrobacterium* harboring an all-in-one vector carrying an sgRNA targeting the nearby S176 and P180 in the *NtEPSPS-B* gene, Cas9, nptII expression cassettes, and a GT donor template 1.0 kb *NtEPSPS-B* coding region with mutations (T176I and P180S) with or without RS-1 treatment. We isolated GT callus lines by CAPS analysis (P180S mutations carry a new *Hin*dIII restriction enzyme recognition site). CAPS-positive bands were detected in one (NtEPSPS_GT DMSO_1) and two (NtEPSPS_GT RS-1_1 and 2) independent lines from 384 transgenic calli treated with DMSO and RS-1, indicating that GT frequency was 0.26% and 0.52% in the treatment of DMSO and RS-1, respectively (Supplementary Table 4). PCR products amplified from CAPS-positive calli were cloned and sequenced to confirm the introduction of TIPS mutations in the *NtEPSPS-B* gene even at low efficiency (Supplementary Table 5). Unfortunately, mutant plants that carried the TIPS mutations in *NtEPSPS-B* gene *via* GT could not be regenerated from these CAPS-positive callus lines.

## Discussion

Positive–negative selection to enrich GT cells has been developed and applied successfully to the modification of several endogenous target genes in rice *via* GT (Terada et al., 2002; Yamauchi et al., 2009, 2014; Ono et al., 2012; Nishizawa-Yokoi et al., 2015). Using this method, because transgenic cells carrying T-DNA that had integrated randomly could be excluded by expression of the negative selection marker, no transgenes were left in the host genome other than at the target locus. However, to date, GT with positive–negative selection has been applied exclusively in rice. In addition, it is difficult to construct the large GT vector required, which has two negative selection marker cassettes at both ends of the vector and a positive selection marker cassette within a 6-kb DNA donor template, in which a long homology arm is needed for HR between the GT vector and target locus by use of spontaneous DSBs. In contrast, our novel all-in-one vector comprises a conventional CRISPR/Cas9 vector with an extra 1-kb DNA donor template; thus, unlike GT with positive–negative selection, long PCRs (>3–4 kb) are not needed to identify GT candidates *via* the CRISPR/Cas9-mediated GT method. The CRISPR/Cas9-mediated GT method developed in the present study provides a very simple and user-friendly approach. In addition, we attempted to establish an efficient CRISPR/Cas9-mediated GT system using a combination of an all-in-one vector and treatment with the small-molecule RS-1, which is known to enhance HR in mammalian cells. Evaluation of the ratio of GT callus to transgenic callus carrying the randomly integrated all-in-one vector revealed that the GT frequency under treatment with RS-1 was slightly, but consistently, higher than that of controls in both rice and tobacco. Although the impact of treatment with RS-1 on GT efficiency was limited even in rice and tobacco, our findings suggest that RS-1 has the potential to improve the frequency of CRISPR/Cas9-mediated GT in plants. Whereas, we also tested the effect of SCR7—a DNA ligase 4 inhibitor—on GT efficiency in rice, there were no significant differences with and without SCR7 treatment (data not shown). Structural optimization of RS-1 to plant Rad51, or structural modification of RS-1 to enhance delivery efficiency into plant cells, is expected to lead the development of more effective approaches to establish a universal GT system for various types of crops.

In tobacco, we found that CRISPR/Cas9-mediated additional mutations were also generated at the target site in T_1_ progenies with the all-in-one vector, but not in siblings in which the all-in-one vector was segregated out (Supplementary Figure 4). These results suggest that Cas9-mediated DNA cleavage occurred in tobacco somatic cells even after the introduction of a single point mutation *via* GT within the seed region of the target sequence. To completely abolish induction of these *de novo* mutations in T_1_ tobacco plants, it is essential that multiple mismatches, or a single mismatch, are introduced into the sgRNA target site or PAM sequence *via* GT, at least in dicot plants.

CRISPR/Cas9-mediated GT strategies have been applied for precise gene modification in plants by several research groups. Geminivirus-based replicons have been used for transient expression of SSNs and delivery of DNA donor template, resulting in successful knock-in of reporter or antibiotic-resistance genes, etc., into an endogenous target gene in tobacco (Baltes et al., 2014), tomato (Cermák et al., 2015), potato (Butler et al., 2016), cassava (Hummel et al., 2018), rice (Wang et al., 2017), and wheat (Gil-Humanes et al., 2017). Puchta and colleagues (Fauser et al., 2012; Schiml et al., 2014) developed an *in planta* GT strategy in which DSBs were induced by SSNs at both ends of a GT donor template on a GT vector integrated into the host genome and at an endogenous target gene, resulting in repair of the target gene *via* HR using a released linear GT donor from a GT vector in *Arabidopsis*. Using these strategies, although GT frequency was still low in higher plants, GT cells were enriched by counter selection derived from the introduction of targeted point mutations conferring resistance to herbicide, knock-in of antibiotic resistance or a reporter gene into the target gene *via* GT. Miki et al. (2018) revealed that not only in-frame reporter gene knock-in but also the introduction of amino acid substitutions, i.e., non-selectable traits, into the endogenous target genes was achieved by the expression of Cas9 under the egg cell-specific promoter in *Arabidopsis*. This tool provides a powerful, but thus far *Arabidopsis*-specific, approach.

Here, we have established a CRISPR/Cas9-mediated GT strategy using an all-in-one vector in combination with RS-1 treatment in rice and tobacco. Although removal of an all-in-one vector remains challenging in vegetative propagation of plants, we are hopeful that our approach will become widely applicable for precise genome modification in a variety of crops. Therefore, we are currently screening several chemicals with the aim of improving GT efficiency in plants.

## Data Availability Statement

The raw data supporting the conclusions of this article will be made available by the authors, without undue reservation.

## Author Contributions

AN-Y designed the research and wrote the manuscript. AN-Y and MM conducted the experiments. ST commented on the research and edited the manuscript. All authors contributed to the article and approved the submitted version.

## Conflict of Interest

The authors declare that the research was conducted in the absence of any commercial or financial relationships that could be construed as a potential conflict of interest.
