# Supplementary material for: A Universal System of CRISPR/Cas9-Mediated Gene Targeting Using All-in-One Vector in Plants
Source: Front Genome Ed. 2020 Nov 25;2:604289. doi: 10.3389/fgeed.2020.604289 (PMC8525384; doi:10.3389/fgeed.2020.604289)
Supplement: Supplementary file 1 [file Data_Sheet_1.docx]

**
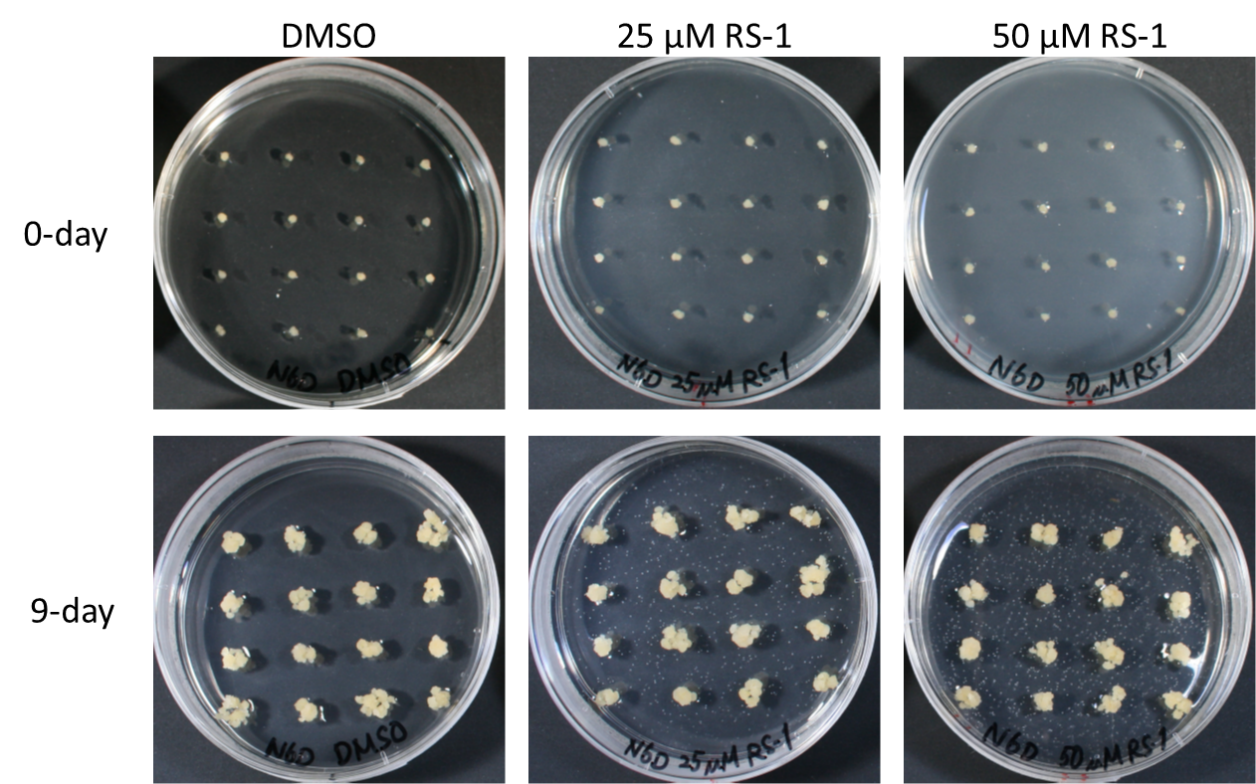
Supplementary Figure 1. The effect of the treatment with RS-1 on the growth of rice calli**

Three-week old rice calli were transferred to medium containing DMSO (control; left panels), 25 µM RS-1 (middle panels) or 50 µM RS-1 (right panels) and were cultured for 9 days.


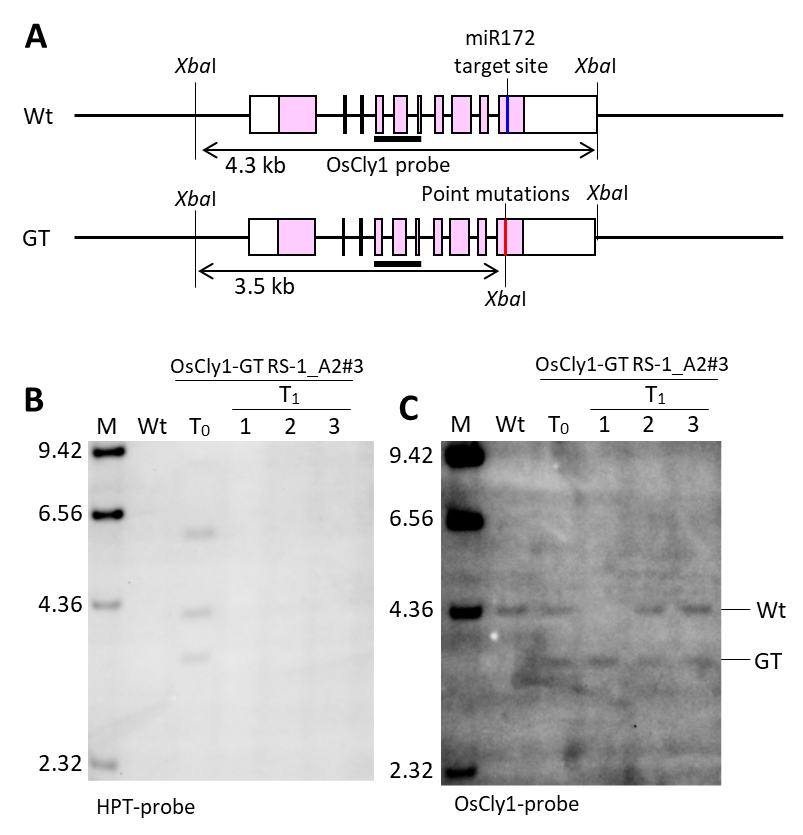


**Supplementary Figure 2.** **Progeny plants carrying modified *OsCly1* gene and lacking all-in-one GT vector can be obtained via segregation by crossing.**

A, Genomic structure of the wild-type *OsCly1* locus (top) and the modified *OsCly1* locus in GT plants (bottom). B, Southern blot analysis with the hpt- (left) and *OsCly1*-specific probe (right) using *Xba*I-digested genomic DNA of wild-type, T_0_ regenerated plants, and T_1_ progeny of OsCly1-GT RS-1_A2#3. Line no. 1 of T_1_ plant was found to carry modified *OsCly1* via GT in biallelic state and lack all-in-one vectors.


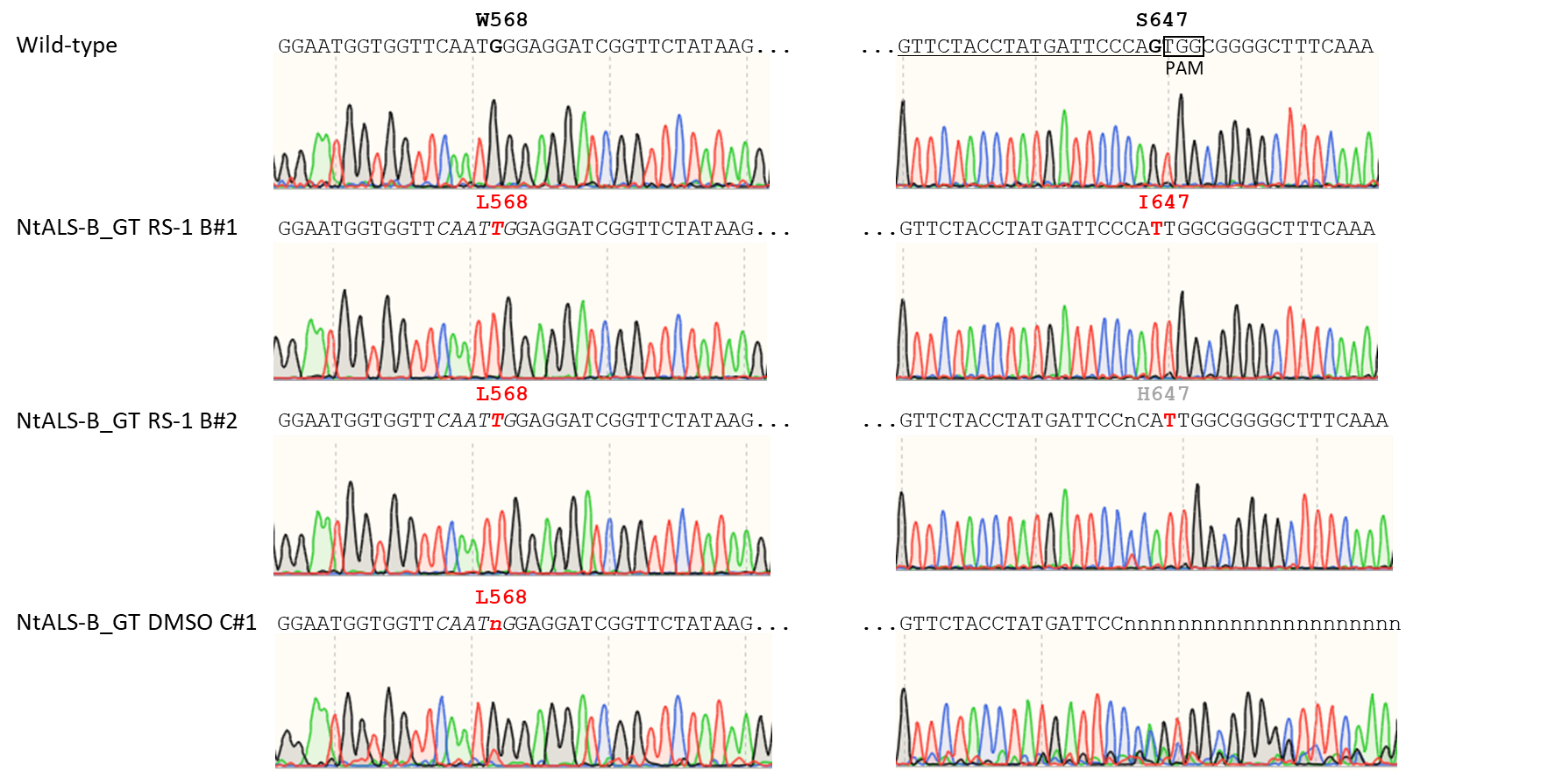


**Supplementary Figure 3. Sequencing chromatograms of mutation sites in the NtALS-B gene in wild-type and NtALS-B_GT T_0_ plants.**

Direct sequencing of PCR fragments derived from T_0_ plants of NtALS-B_GT RS-1 B#1 and B#2 revealed that W568L/S647I mutations via GT were induced in the NtALS-B locus in the biallelic state. However, additional CRISPR/Cas9-mediated mutations were generated at the target site as a chimeric state in T_0_ plants of NtALS-B_GT RS-1 B#2 even after the introduction of S647I mutations via GT. In T_0_ plants of NtALS-B_GT DMSO C#1, both GT- and CRISPR/Cas9-mediated mutations occurred in a chimeric state.


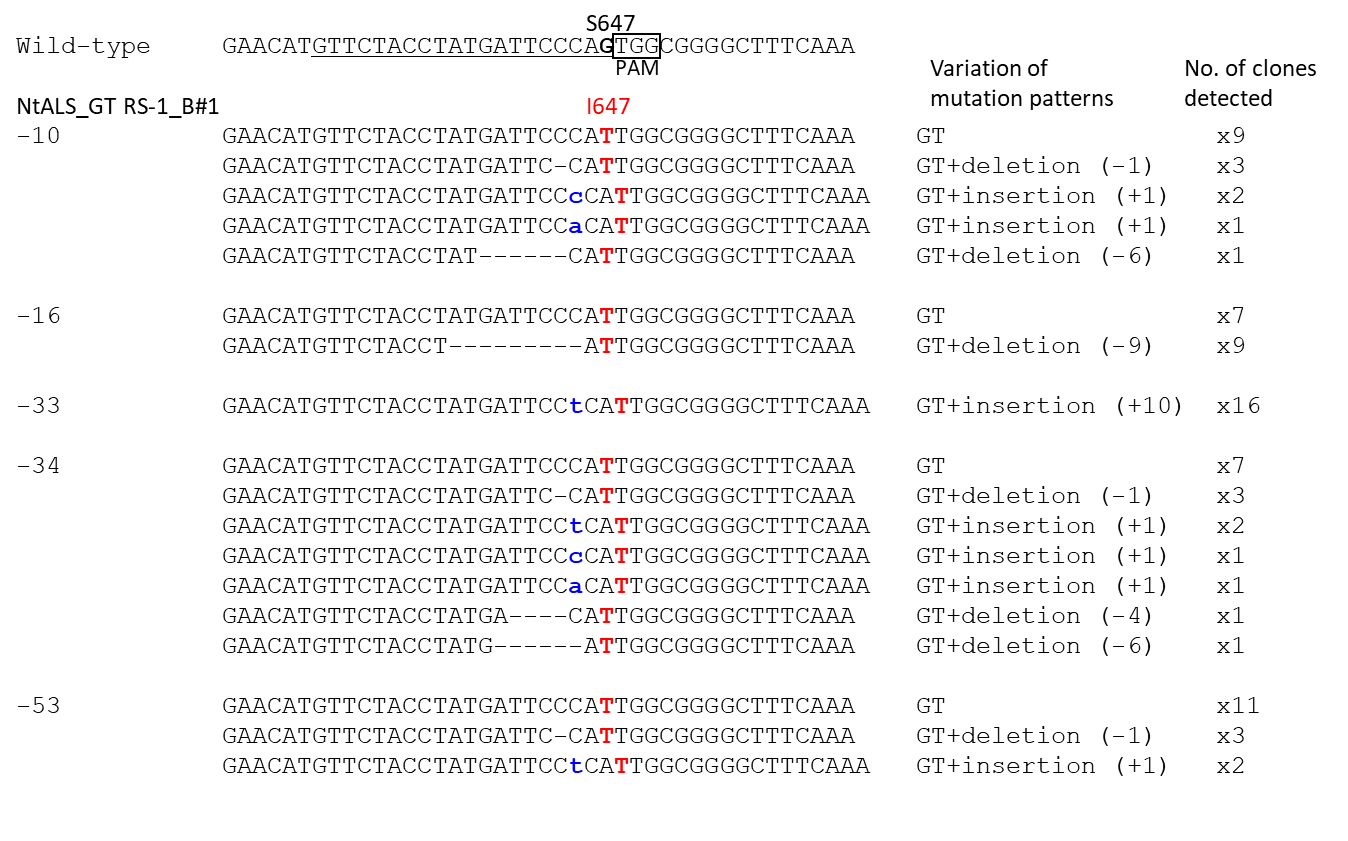


**Supplementary Figure 4. Diverse patterns of CRISPR/Cas9-mediated additional mutations were found in T_1_ plants of NtALS-B_GT RS-1 B#1.**

PCR products were cloned into cloning vector and 16 clones were sequenced in each line using a universal primer. Target sites of sgRNA are underlined, point mutations introduced by GT are in red, and the insertional mutations are noted in blue.


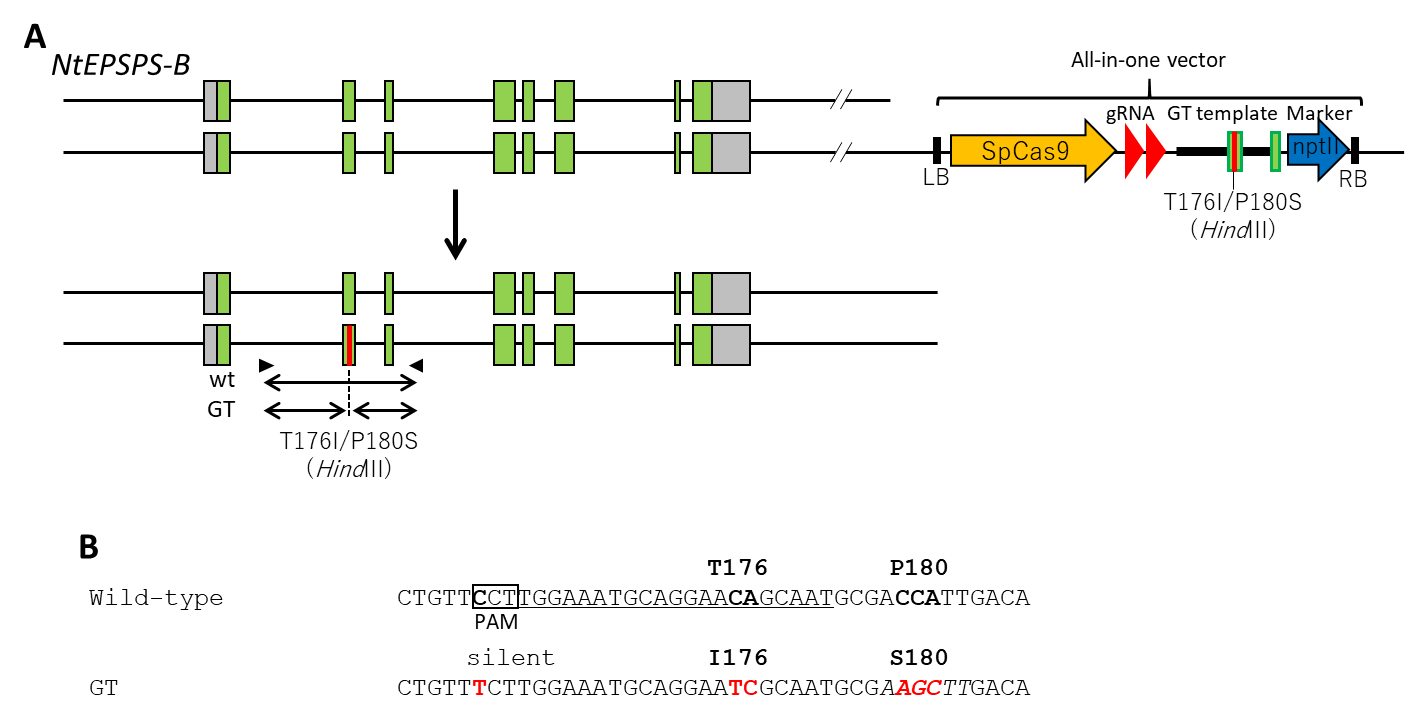


**Supplementary Figure 5. The introduction of point mutations into the tobacco *EPSPS-B* (*NtEPSPS-B*) locus via CRISPR/Cas9-mediated GT with all-in-one GT vectors.**

A, (Top) The genomic structure of the wild-type *NtEPSPS-B* locus and schematic diagram of all-in-one GT vector targeting the *NtEPSPS-B* locus. (Bottom) The genomic structure of the modified *NtEPSPS-B* locus in GT plants. Black arrows indicate the primer set for CAPS analysis to identify the GT lines. B, Nucleotide sequence of wild-type (top) and modified (bottom) *NtEPSPS-B* locus via GT. Target sites of sgRNA are underlined, point mutations introduced by GT are in red, and the recognition sites of *Hin*dIII (AAGCTT) used for CAPS analysis are in italics.

Supplementary Table 1 List of primers used in this study

Supplementary Table 2 Frequency of modified OsALS gene with targeted mutations (W548L/S627I) via GT in CAPS-positive calli.

Supplementary Table 3 Transmission of mutations that occurred via GT in transgenic calli to regenerated plants.

Supplementary Table 4 Summary of GT experiments targeting NtEPSPS-B gene using all-in-one GT vector

GT positive callus lines were designated as follows: *, NtEPSPS_GT DMSO#1; **, NtEPSPS_GT RS-1#1 and #2.

Supplementary Table 5 Frequency of modified NtEPSPS-B gene with targeted mutations (T176I/P180S) via GT in CAPS-positive calli.
